# Reactive astrocytes in prion diseases: Friend or foe?

**DOI:** 10.1371/journal.ppat.1012286

**Published:** 2024-06-20

**Authors:** Natallia Makarava, Rajesh Kushwaha, Ilia V. Baskakov

**Affiliations:** 1 Center for Biomedical Engineering and Technology, University of Maryland School of Medicine, Baltimore, Maryland, United States of America; 2 Department of Neurobiology, University of Maryland School of Medicine, Baltimore, Maryland, United States of America; National Institutes of Health, UNITED STATES

## Are reactive astrocytes protective or detrimental?

**Fig 1:**
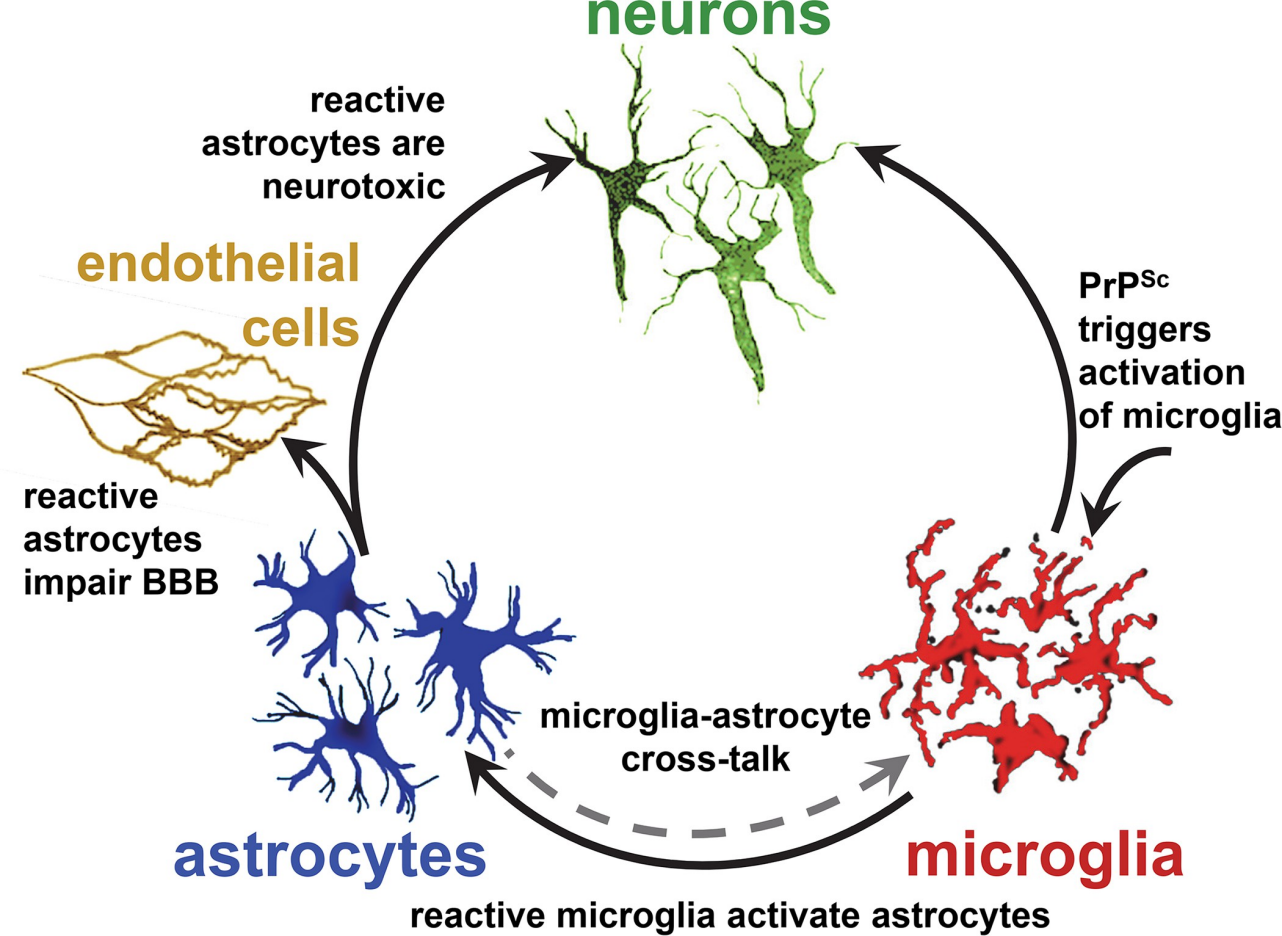
Schematic diagram illustrating interaction between microglia, astrocytes, endothelial cells, and neurons in prion diseases. PrP^Sc^ triggers proinflammatory phenotype in microglia [40]. Reactive microglia activate astrocytes [7]. In reactive state, astrocytes lose homeostatic functions that support neurons [9]. Reactive astrocytes associated with prion disease are neurotoxic [7] and have deleterious effects on endothelial cells of BBB [8].

In the healthy brain, astrocytes play vital roles essential for neuronal transmission and blood–brain barrier (BBB) integrity. The transformation of astrocytes into reactive states constitutes a biological response of the central nervous system (CNS) to insults and changes in brain environment. It is well known that astrocytes can replicate and accumulate prions independently of neurons [1–5]. However, considerably less is known about the impact of their reactive transformation on neuronal function and neurodegeneration. In prion diseases, the beneficial role of reactive astrocytes appears to be linked to astrocyte-produced milk fat globule epidermal growth factor 8 (Mfge8), which facilitates the engulfment of apoptotic bodies and the clearance of cellular debris [6]. However, alongside the enhancement of protective functions, it is crucial to weigh potential deficits in homeostatic roles and the emergence of detrimental functions when evaluating the overall impact of reactive astrocytes on disease progression. Recent studies suggested that reactive astrocytes may exert a net negative impact on both neurons and endothelial cells. Reactive astrocytes isolated from prion-infected animals exhibit detrimental effects on primary neurons causing reduction in dendritic spine size and density along with impairment of synapse integrity [7] (Fig 1). The synaptotoxic effects were mediated by changes in the astrocytic secretome, highlighting the potential role of altered signaling pathways in neuronal dysfunction. In addition to their effects on neurons, reactive astrocytes disrupt the integrity of the BBB. Coculturing experiments involving astrocytes from prion-infected animals or exposure to conditioned media from reactive astrocytes induced a disease-associated phenotype in endothelial cells isolated from normal mice [8] (Fig 1). This phenotype was characterized by the down-regulation and aberrant localization of tight and adherens junction proteins, and an increase in permeability of endothelial layers. Notably, a very strong reverse correlation was observed between the degree of astrocyte activation and the incubation time to the prion diseases [9]. Animal groups with rapid disease progression exhibit more severe astrocyte reactivity, suggesting a potential link between phenotypic changes in astrocytes and disease severity. This observation brings up the possibility that phenotypic changes in reactive astrocytes contribute to the faster disease progression. Consistent with this hypothesis, inhibition of the unfolded protein response, which is exacerbated in reactive astrocytes, via selective targeting of PERK signaling, was found to prolong the incubation time to terminal disease in mice [10]. In summary, reactive astrocytes associated with prion diseases exhibit harmful effects on neurons and endothelial cells and are likely contributors to disease progression. Further elucidation of the underlying mechanisms driving astrocyte reactivity may hold therapeutic potential for mitigating neurodegenerative processes associated with prion diseases.

## What do we know about reactive phenotype of astrocytes in prion diseases?

In adults, astrocytes exhibit functional diversity, giving rise to distinct neural circuit–specialized subpopulations. Single-cell transcriptome analysis of mouse brains has identified 7 subtypes of astrocytes that are developmentally predetermined and regionally specified [11]. Subpopulations of astrocytes are interconnected via gap junctions, forming networks that respond to stimuli in uniform manners. It is not surprising that astrocytes respond to prion infection in a region-specific manner [9,12,13]. Unexpectedly, transcriptome analysis has revealed a significantly more profound response of astrocytes to prion infection compared to neurons [14–16]. This response perturbs multiple astrocyte-specific functions, including BBB regulation, transporter activity, myelination, energy metabolism, ion channels, extracellular matrix composition, growth factors, and neuroprotection [9]. Of particular interest, reactive astrocytes down-regulate genes involved in neuronal support and synapse formation and maintenance [9]. Moreover, there is a marked down-regulation of ribosomal and mitochondrial proteins, including components of the electron transport chain, suggesting a deficit in energy production [15]. Upon transitioning to reactive states, astrocytes lose polarization and retract end-feet from blood vessels as evident from a drastic change in localization of Aqp4, the most prevalent water channel essential for maintaining water homeostasis in the CNS [8]. Live-cell imaging has demonstrated a loss of phagocytic activity in reactive astrocytes isolated from prion-infected animals [17]. In summary, in prion diseases, reactive astrocytes lose homeostatic functions essential for neuronal support, giving rise to a detrimental, neurotoxic phenotype.

## Do astrocytes respond to prions in a strain- and/or region-specific manner?

Prion strains display distinct affinities for different brain regions and follow unique timelines of disease progression. Transcriptome analysis of mice exposed to 4 prion strains reveals a consistent astrocytic reactive profile within individual brain regions across all strains [9]. However, the degree of astrocyte activation within individual regions varies, influenced by the strain-specific regional tropism and progression timeline. Despite differences in cellular preferences among the strains, there is significant overlap in differentially expressed astrocytic gene sets, underscoring a shared reactive phenotype regardless of whether prion strains replicate in neurons or astrocytes [9]. Further investigation is necessary to determine the extent to which the reactive phenotype of astrocytes in prion-infected mice mirrors that of human prion diseases. Consistent with the astrocytic response observed in mice, astrocytes in sporadic Creutzfeldt–Jakob disease exhibit an up-regulation of C3, a marker associated with a neurotoxic phenotype [18].

**Fig 2:**
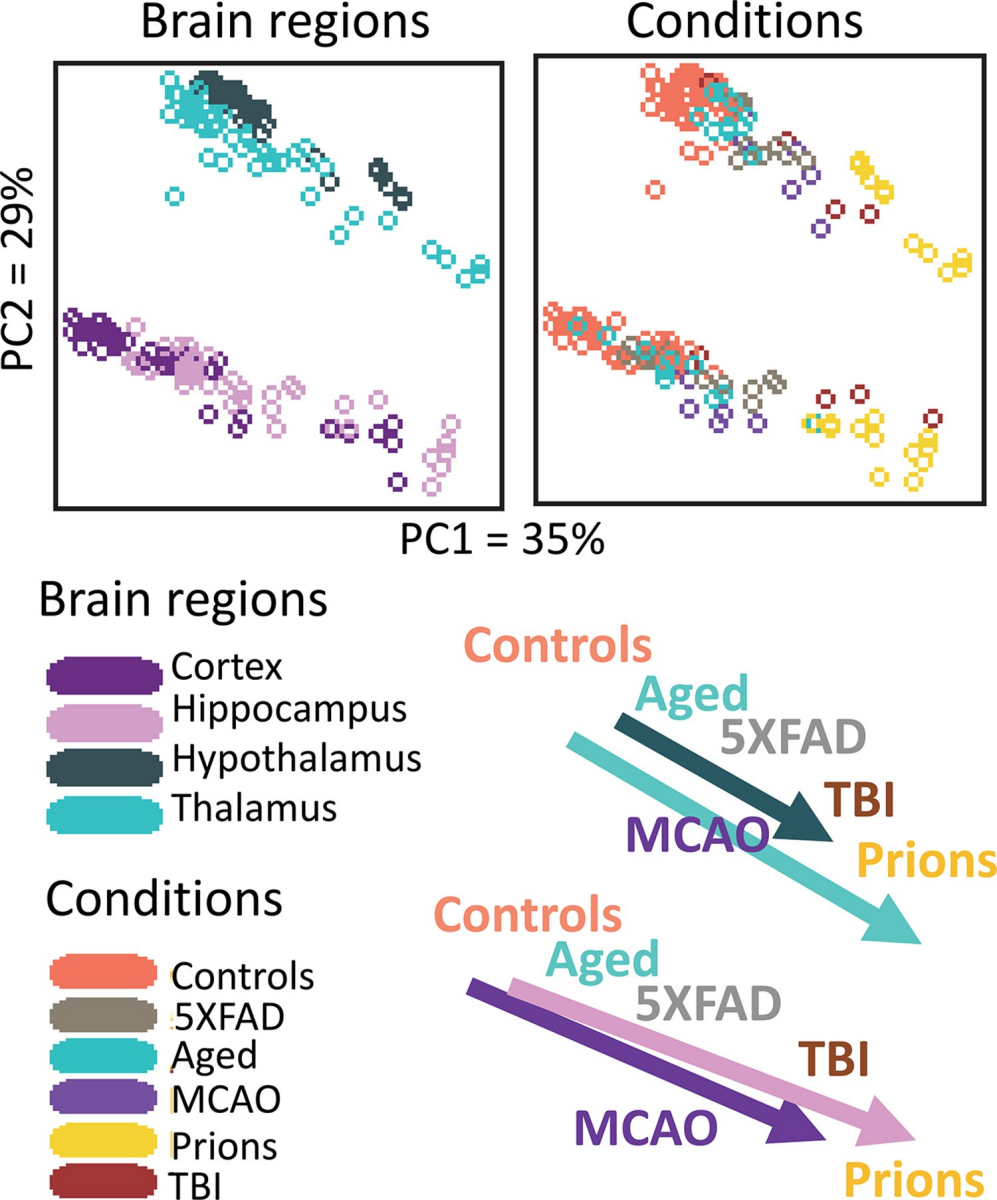
Principle component analysis (PCA) of region-specific differences in expression of astrocyte specific across 5 animal groups: prion-infected mice, mice subjected to TBI, 5XFAD mouse model of Alzheimer’s disease, mice subjected to ischemic insult (MCAO, middle cerebral artery occlusion), and aged 24-month-old mice. Cortex, hippocampus, thalamus, and hypothalamus were analyzed in each group. PCA revealed well resolved continuums of astrocytic phenotypes shown on bottom right: one shared by the cortex and hippocampus and another by the thalamus and hypothalamus. Each dot represents an individual animal. The figure was adapted from [21].

The region-specific homeostatic identity shapes the astrocyte reactive phenotype elicited in response to prion infection [9,12,13]. The observations in prion disease resonate with findings from studies on other neurodegenerative conditions, demonstrating that astrocytes respond to insults in a region-specific manner while maintaining their distinct identities within those regions [19,20]. Moreover, profiling of reactive astrocytes in mice across various conditions, including prion infection, traumatic brain injury (TBI), the 5xFAD model of Alzheimer’s disease, ischemic insult, and normal aging, has revealed that insult-specific phenotypes do not segregate neatly from each other but instead exhibit partial overlap, forming continua of phenotypes (Fig 2) [21]. Notably, these continua of phenotypes are region-specific. These findings suggest that, in defining reactive phenotypes, the region-specific homeostatic identity of astrocytes is likely to be as significant as the nature of the insult.

## Do reactive microglia and astrocyte talk to each other?

Recent years have unveiled intricate cross-talk between microglia and astrocytes [22,23]. The reactive states of both cell types appear to be mutually dependent and regulated through multiple signaling pathways [22,23]. Cross-comparison of several brain regions in animals infected with different prion strains revealed that the degrees of microglia and astrocyte activation follow the same ranking orders, suggesting that their reactive states associated with prion diseases are coordinated via cross-talk [9,16,24]. Indeed, upon exposure to cell media conditioned by reactive microglia isolated from prion-infected animals, primary astrocytes acquired a neurotoxic phenotype characterized by hypertrophic morphology and down-regulation of genes responsible for synaptogenic functions [7] (Fig 1). In experiments on LPS-activated microglia, proinflammatory factors TNFα, IL1α, and C1q secreted by microglia were found to induce a neurotoxic phenotype in astrocytes [25]. However, contrary to expectations, triple TNFα^−/−^IL1α^−/−^C1q^−/−^ knockout accelerated the progression of prion diseases, questioning the role of microglia-secreted TNFα, IL1α, and C1q as universal drivers of the neurotoxic astrocytes [26]. Furthermore, depletion of microglia resulted in an exacerbated reactive phenotype in astrocytes and accelerated progression of prion disease [27–29]. The above results, while supporting the idea of cross-talk, suggest that astrocytes might compensate for the lack of reactive microglia via an exuberated proinflammatory response. Do reactive astrocytes influence the phenotype of microglia? In the reactive states, including those associated with prion diseases, astrocytes up-regulate the expression of IL-33 and C3 [7], the drivers of microglia-mediated phagocytosis and elimination of synapses [30,31]. To summarize, suppressing or altering the reactive state of one cell type will likely change the reactive phenotype of another cell type. While the reactive states of both astrocytes and microglia are mutually dependent, the mechanistic details of their cross-talk in prion diseases remain poorly understood.

## Is astrocyte reactivity reversible?

Phenotypic changes in astrocytes have long been considered irreversible. Nevertheless, reactive astrocytes isolated from injured spinal cords reverted their phenotype upon transplantation into a naïve spinal cord [32], suggesting that the preservation of reactive phenotypes relies on persistent stimuli or the presence of environmental factors. In neurodegenerative diseases, the STAT3 transcription factor has been proposed to serve as a master regulator of astrocyte reactivity [33,34]. Selective inhibition of the STAT3 pathway in astrocytes suppressed their activation or reversed their reactive phenotype, improving disease outcomes in animal models of Alzheimer’s and Huntington’s diseases [35–37]. Activation of STAT3 was also observed in animals infected with prions [38]. However, its role in driving astrocyte reactive states associated with prion diseases has not yet been examined. Nevertheless, STAT3 represents one of the main targets for reversing the reactive phenotype of astrocytes. An alternative strategy involves targeting the compartmental localization of cyclic adenosine monophosphate (cAMP). In optic nerve injury, a balance between neurotoxic, C3-positive, and neuroprotective, C3-negative reactive astrocyte populations was shown to be regulated by distinct pools of compartmentalized cAMP [39]. Raising nuclear or depleting cytoplasmic cAMP in reactive astrocytes promoted retinal ganglion cell survival. However, it remains to be determined whether this strategy is suitable for a broad spectrum of neurodegenerative diseases. Moreover, since astrocytes respond to prions in a region-specific manner, it might be challenging to manipulate their reactive phenotype uniformly across the whole brain.
